# Automated fluid monitoring to optimize the follow-up of neovascular age-related macular degeneration patients in the Brazilian population

**DOI:** 10.1186/s40942-025-00695-0

**Published:** 2025-07-06

**Authors:** Virginia Mares, Gregor S. Reiter, Aniel Feitosa, Markus Gumpinger, Hrvoje Bogunovic, Ursula Schmidt-Erfurth, Marcio B. Nehemy

**Affiliations:** 1 Laboratory for Ophthalmic Image Analysis, Department of Ophthalmology and Optometry, Medical University of Vienna, Währinger Gürtel 18-20, Vienna, 1090 Austria; 2 Department of Ophthalmology, Federal University of Minas Gerais, Belo Horizonte, Brazil

**Keywords:** Anti-VEGF therapy, Artificial intelligence, Biomarker detection, Deep learning, Disease activity prediction, Multimodal retinal imaging, Neovascular age-related macular degeneration, Optical coherence tomography, Retinal fluid segmentation

## Abstract

**Objectives:**

To investigate the efficacy of an artificial intelligence (AI)-based fluid monitoring tool in optimizing the monitoring of neovascular age-related macular degeneration (nAMD) patients in a Brazilian cohort.

**Methods:**

This is a retrospective real-world study performed in a tertiary center in Brazil, including patients with nAMD. Spectral-domain optical coherence tomography (Spectralis, Heidelberg Engineering, Germany) images were processed at baseline and over 2 years of follow-up. Demographic and clinical data were collected. A deep learning algorithm (Fluid Monitor, RetInSight, Austria) was used to automatically quantify intraretinal fluid (IRF), subretinal fluid (SRF) and pigment epithelial detachment (PED). A longitudinal panel regression model and Log-Rank test were performed to assess the correlation between fluid volumes and treatment frequency, visual outcomes, macular atrophy (MA) and subretinal fibrosis (SF) development.

**Results:**

Ninety-nine eyes from 84 patients were included. Fifty-eight eyes were treatment-naïve. Higher IRF and PED in the 6 mm area were correlated with worse visual outcomes over a 2-year follow-up (*p* = 0.01 and *p* < 0.001, respectively). Higher IRF, SRF and PED were correlated with an increased risk of SF development (*p* < 0.001, *p* = 0.049 and *p* = 0.02 respectively). MA development showed no significant correlation with higher IRF, SRF nor PED in this analysis. Higher SRF volume correlated with a greater number of required intravitreal injections over 2-years.

**Conclusion:**

This study investigates the multifaceted landscape of nAMD in a tertiary center in the Southeast Brazil using an AI-based fluid monitoring tool. Further studies that highlight the significance of using newly validated technologies across diverse populations worldwide will be of interest.

## Introduction

Age-related macular degeneration (AMD) stands as one of the leading cause of irreversible vision impairment worldwide, particularly affecting individuals aged 50 and older [1, 2]. Among its subtypes, neovascular AMD (nAMD) presents a major threat to vision due to the rapid progression and for its macula targeting location. Macular neovascularization is responsible for fluid leakage into intraretinal and subretinal space and the advent of intravitreal anti-VEGF therapy was a paradigm shift for controlling disease activity [3, 4]. Optical coherence tomography (OCT) has become the primary imaging device for detecting and monitoring retinal fluid [5]. A retrospective study performed in a large tertiary center in London, the Moorfields Eye Hospital, showed more than 10-fold increase in numbers of anti-VEGF injections from 2009 to 2019, with nAMD being the foremost condition on injections demand, and consequently, the inherent costs [6]. In Brazil, the prevalence of AMD and its treatment mirrors the global trend. A previous study using the Brazilian public health system database showed an increase of 1.088% in prevalence of anti-VEGF injections in the country from 2010 to 2019 [7]. Yet, anti-VEGF injections represent an important burden to public and supplementary health care systems worldwide, and with the ageing population and the advent of anti-complement intravitreal therapy for geographic atrophy the numbers may rise significantly [8, 9]. 

Brazil is a multi-ethnic continental country with over 200 million people and is one of the few countries worldwide to offer a free universal coverage health care system to all its citizens, covering from primary to tertiary care [10]. More than 75% of the population relies exclusively on this system to access clinical appointments, exams, and surgical procedures, including ophthalmology care [11]. Furthermore, the distribution of eye care professionals in the country is not uniform across the vast and diverse population. The last census reported by the Brazilian Ophthalmology Council (CBO) from 2021 showed a clear concentration of eye doctors in the southeast region, with a median of 20.2 ophthalmologists per 100,000 inhabitants, while in the northern region, for example, the median density was 6.9 ophthalmologists per 100.000 inhabitants [12]. The dynamic nature of retinal diseases such as nAMD demands close monitoring of patients to detect early signs of disease recurrence, treatment demand, and/or progression to late stages such as macula atrophy (MA) and subretinal fibrosis (SF), which can be challenging to patients and to the health system [13]. Yet, a combination of large population, unbalanced resources and high monitoring demand may pose a significant challenge to the eye care health system and professionals in a daily practice.

In the past recent years, there has been a notable increase of artificial intelligence (AI)-based tools capable of identify and quantify retinal imaging biomarkers [14]. The integration of deep learning algorithms in AMD patients follow-up is promising for enhancing decision-making processes and optimizing treatment strategies [15–17]. One limitation of the recent published studies regarding AI-based technology is the predominance of Caucasian population in validating and testing the algorithms, which can lead to a lack of generalization of the results. The Brazilian society was initially formed by a confluence of European, African and Indigenous ethnicities [18]. Currently, according to the Brazilian Institute of Geography and Statistics (IBGE), the population can be classified, based on ethnicity as white, black, brown (multiracial), indigenous and yellow (east Asian). The purpose of this study is to investigate the use of an AI-based medical device regulation (MDR)-approved fluid monitoring tool to optimize nAMD patients control in a real-world Brazilian cohort.

## Subjects and methods

### Participants

This is a retrospective study performed in a tertiary center in Minas Gerais, Brazil, including patients with nAMD treated in a treat-and-extend regimen with Aflibercept or Ranibizumab over 2 years of follow-up. The study was conducted in compliance with the declaration of Helsinki and had approval from the institutional review board (CAAE 58850622.4.0000.5149). Patients’ informed consent was exempted because of the retrospective nature of this study using fully anonymized retinal images. Electronic medical records regarding age, sex, best-corrected visual acuity (BCVA), number of injections, and presence of MA or SF were collected. Anonymized spectral-domain (SD)-OCT (Spectralis, Heidelberg Engineering, Germany) images were segmented at baseline and every visit during 2 years of follow-up. Patients with ungradable scans, less than 1 year follow-up, visual acuity worse than 20/200 in Snellen chart at baseline, history of cataract surgery during the follow-up period, concomitant retinal sight-threatening disease such as advanced glaucoma, epiretinal membrane or vitreo-macular traction, and/or presence of central MA or SF at baseline were excluded. The OCT volumes were not standardized, however the analysed images had at least 19 B-scans per volume. To evaluate the impact of retinal fluid volumes in each compartment at baseline in the number of injections and final BCVA the patients were divided into two groups. The highest 25% quartile of fluid volume in each compartment (intraretinal fluid [IRF], subretinal fluid [SRF], and pigment epithelial detachment [PED]) at baseline in the central 6 mm were classified as high fluid volume subgroup. The remaining 75% of patients were classified as low fluid volume subgroup for each respective fluid compartment [19–21]. 

### Visual acuity, macular atrophy and subretinal fibrosis determination

BCVA was tested every visit using Snellen chart and converted to early treatment of diabetic retinopathy study (ETDRS) score. MA and SF were graded by the attending physician and extracted from the medical records. This assessment was based on either OCT images, fundoscopy, fundus photography and fundus autofluorescence images when available. Further, all the included baseline OCT images were analysed by a retinal specialist (VM) to exclude baseline central MA and SF, in case it was not mentioned in the chart, for the MA and SF development analysis. Retinal pigment epithelium (RPE) loss with associated retinal ellipsoid zone loss more than 250 μm in diameter detected in the central 3 mm area were excluded as MA. OCT images showing a compact multilaminar hyperreflective material situated above or below RPE, with indistinct RPE layer, in the central 3 mm area was excluded as SF [22]. Subretinal hyperreflective material (SHRM) was not excluded.

### Automated quantification of retinal fluid

**Fig. 1:**
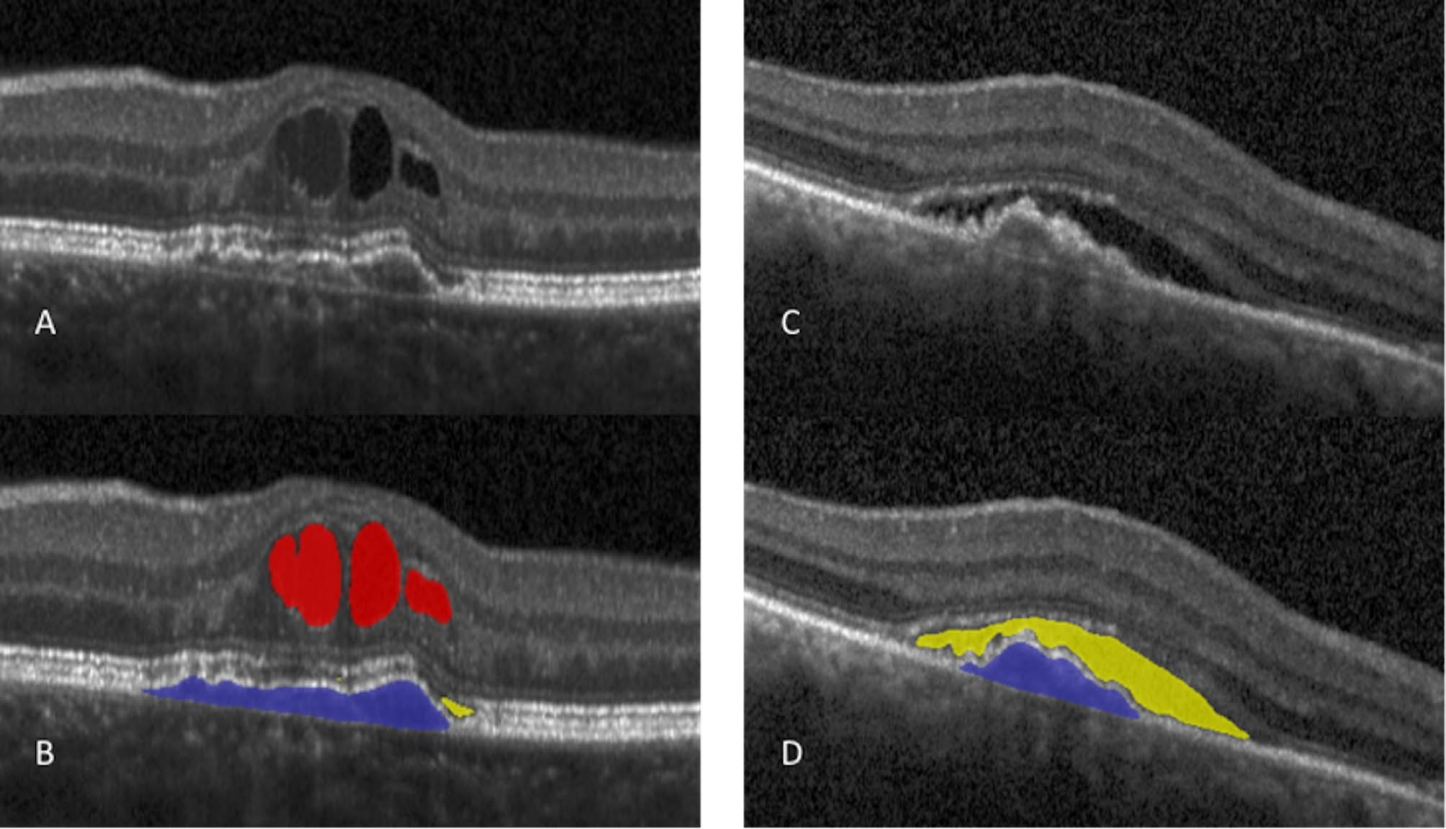
Automated segmentation of intraretinal fluid (red), subretinal fluid (yellow) and pigment epithelial detachment (blue)

IRF, SRF and PED volumes within the central 1 mm and 6 mm macular subfields were automatically segmented and quantified using the MDR-certified deep learning algorithm (Fluid Monitor, RetInSight, Vienna, Austria) in all time points. The algorithm uses a convolutional neural network (CNN) to identify retinal fluid in each compartment and PED on a pixel-level (Fig. 1). The number of assigned pixels can be computed into an estimation of fluid volumes in nanoliters (1 nL = 0.001 mm [3]) and their respective compartments [23, 24]. 

### Statistical analysis and fluid function correlation

Statistically significant Lagrange Multiplier Breusch-Pagan and Chow test demonstrated that a longitudinal time-series data analysis would be better suited to correlate fluid volume in each compartment with BCVA over a 2-year follow-up. Panel regression with fixed or random effect model was chosen based on Hausman and Sargent-Hansen chi-squared test for each variable. A Log-Rank test with Kaplan Meier curve was used to analyse the correlation between fluid volumes in both subgroups in each compartment and new onset MA and SF, as well as MA and SF development over treatment-naïve and pre-treated patients. Spearman correlation was used to analyse the association between fluid volume in each compartment at baseline and number of intravitreal injections after 12 and 24 months.

## Results

Ninety-nine eyes from 84 patients with nAMD were included, whereof 56 (66.7%) patients were female, mean age was 76.5 ± 9.8 years and 58 eyes (58.5%) were treatment-naïve (Table 1). In the central 1 mm area fluid volumes measurements showed IRF with a median of 0 (IQR 0-11.26), SRF presented a median of 1.84 (IQR 0-19.49) and PED with a median of 44.91 (IQR 16.66-102.45). In the central 6 mm area, the median of IRF volume was 1.04 (IQR 0.0–61.94), SRF was 66.54nL (IQR 1.98–292.8) and PED achieved 446.24nL (IQR 135.7-1025.6).

**Table 1:** Demographic data from the 99 eyes from 84 patients included in the study

	Variable	Frequency
		*N* %
**Gender**	Male	28 33.3
	Female	56 66.7
**Age**	Mean ± SD	76.5 ± 9.8
**Eye**	Unilateral	69 82.1
	Bilateral	15 17.9
**Treatment-naïve**	Yes	58 58.5
	No	41 41.5
**Injections (12 months)**	Mean ± SD	5.9 ± 2.6
**Injections (24 months)**	Mean ± SD	13.6 ± 4.2
**Macular atrophy development during follow-up**	Yes	16 18.2
	No	72 81.8
**Subretinal fibrose development during follow-up**	Yes	21 23.9
	No	67 76.1

**Fig. 2:**
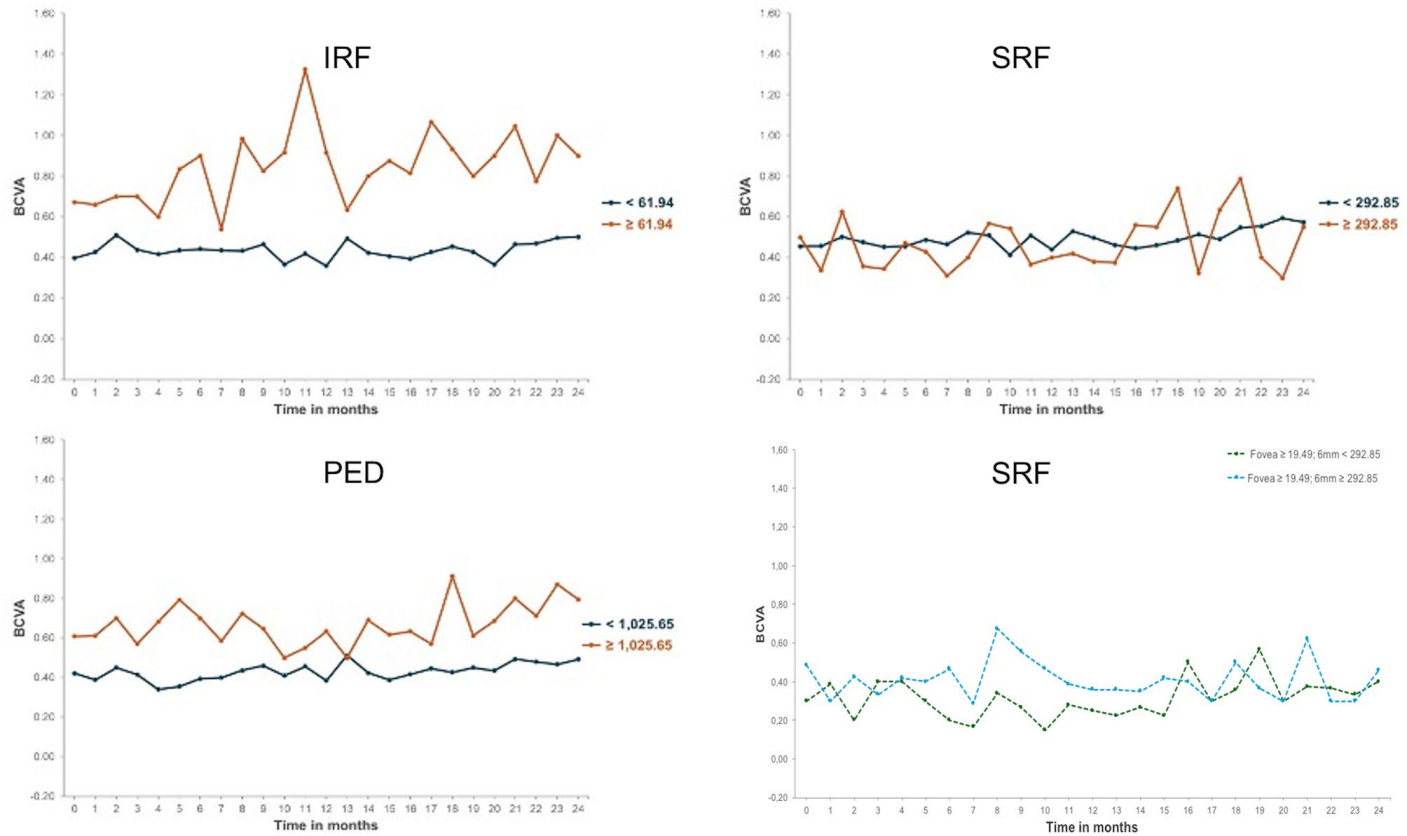
BCVA over time in the higher 25% and lower 75% volume subgroup in the central 6 mm of the macula during follow-up. SRF correlation was statistically significant when fluid volume in the central 1 mm was included in the model. The dotted lines graphic showed that when fluid volumes in the central 1 mm was above 19.49 (Q3) the worst BCVA in the higher fluid volume subgroup (> 292.85nl) was clearer to be seen

The overall behavior of each of the 99 eyes included in this analysis regarding BCVA over the follow-up time is shown in the supplementary material. As demonstrated in the IQR numbers, the highest 25% quartile in each compartment at baseline in the central 6 mm corresponded to eyes with IRF ≥ 61.94nL, SRF ≥ 292.85nL and PED ≥ 1025.65nL. These baseline volumes are higher than reported in a previous analysis from a European cohort [25]. The statistically significant chow test showed that a multivariate regression analysis with fixed timepoints would not be the most appropriate method for assessing visual acuity impairment in this cohort. Therefore, a panel regression analysis was performed showing that higher IRF and PED volumes subgroup in the central 6 mm had a statistically significant impact on the LogMar BCVA measurements, meaning worsening of BCVA over 2 years of follow-up (*p* = 0.01, *p* < 0.001 respectively). Higher SRF volume subgroup was significantly correlated with worse BCVA when fluid volume was also higher than 19.49 (Q3) in the central 1 mm, (*p* = 0.02), as shown in Fig. 2.

**Fig. 3:**
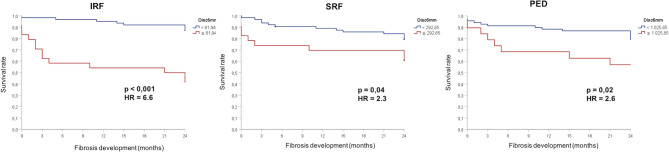
Kaplan-Meier survival curve showing significant higher subretinal fibrosis development in the higher fluid volume subgroup. Hazard ratio (HR), Intraretinal fluid (IRF), subretinal fluid (SRF) and pigment epithelial detachment (PED)

**Fig. 4:**
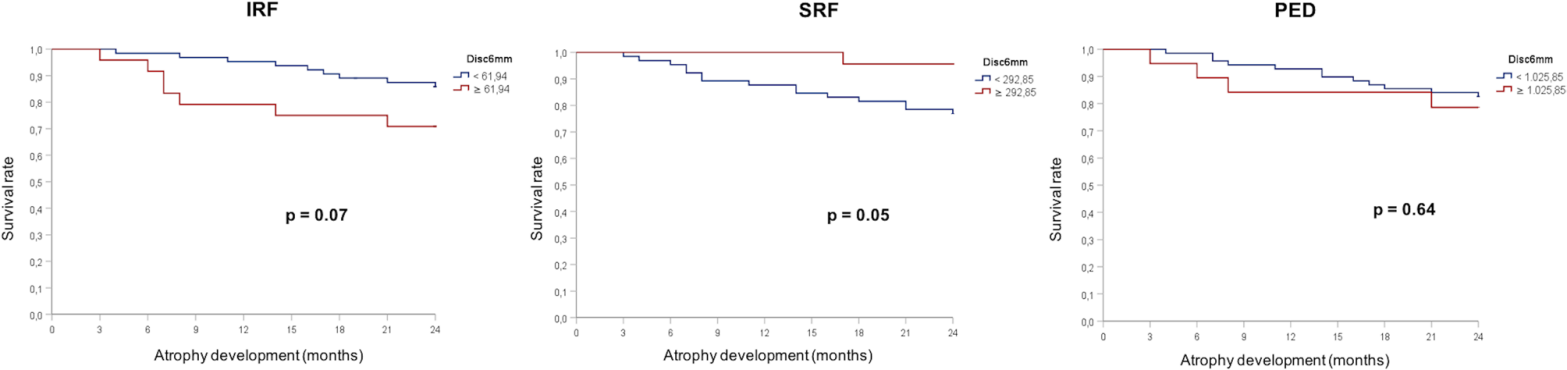
Kaplan-Meier survival curve of macular atrophy development did not show statistically significance difference between the two groups. Intraretinal fluid (IRF), subretinal fluid (SRF) and pigment epithelial detachment (PED)

To further analyse late-stage outcomes, SF and MA development, 11 eyes were excluded after imaging evaluation due to presence of MA or SF in the central 6 mm at baseline. Of 88 eyes, 16 (18.1%) developed MA and 21 eyes (23.8%) developed SF over the 2 years of follow up. The higher IRF and PED subgroups showed increased risk of developing SF in the central 1 and 6 mm of the macula when compared to the lowest 75% subgroups. IRF analysis showed a hazard ratio (HR) of 6.6 (IC95% 2.8–15.5) *p* < 0.001 in the central 1 mm and HR of 6.6 (IC95% 2.8–15.9) *p* < 0.001 in the central 6 mm area of the macula. PED showed HR of 2.5 (IC95%1.1–5.9) *p* = 0.02 for the central 1 mm, and HR of 2.6 (IC95% 1.1–6.1), *p* = 0.02 for the central 6 mm, as shown in Fig. 3. The higher SRF subgroup in the central 6 mm was significantly associated with SF, HR of 2.3 (IC95% 1.0–5.3), *p* = 0.049 but it was not significant in the central 1 mm analysis. Regarding MA, higher fluid volumes did not show statistically significant correlation with its development in any of the compartments (Fig. 4). However, higher IRF (> 61.94nL in the central 6 mm) presented a decreased in average survival time until atrophy development from 22.7 months (95%CI: 21.7–23.7) to 19.8 months (95%CI: 16.8–22.7), *p* = 0.07 in the Log-Rank test. The overlap within the CIs across volume subgroups and *p* > 0.05 associated with a small sample size makes it difficult to determine whether this result is clinically meaningful. However, this result could fit previous findings that strongly correlate type III MNV, which is known to have predominantly more IRF, with MA development [26]. Furthermore, the development of MA and SF showed no significant difference between treatment-naïve and pre-treated patients in this cohort.

Overall, the mean number of injections per eye was 5.9 ± 2.6 over 12 months and 13.6 ± 4.2 over 24 months. An analysis between the total volume of IRF, SRF and PED and treatment needs (number of injections) using Spearman test showed that SRF has a positive, despite weak, correlation on increased number of intravitreal injections after 12 months (*r* = 0.3, *p* = 0.02 in the central 1 mm and *r* = 0.38, *p* < 0.001 in the central 6 mm) and 24 months (*r* = 0.2, *p* = 0.01 in the central 1 mm and *r* = 0.2, *p* = 0.02 in the central 6 mm). IRF and PED were not significantly correlated with the number of injections. When comparing between higher and lower SRF subgroups using a t-test, the results was consistent with previous analysis [20, 27] showing that higher 25% of SRF was associated with higher mean number of injections after 12 months (mean 7.1 ± 2,7, while lower 75% mean of 5.4 ± 2,4) and 24 months (mean 15.0 ± 4.4, while lower 75% mean of 13.0 ± 3.9), *p* = 0.005 and *p* = 0.04 respectively.

## Discussion

AI-based technologies have been described with the potential to bridge some gaps between patients and health care providers with cost-effective and scalable solutions such as automated disease screening, biomarkers segmentation, and risk prediction for late stages outcomes [27–30]. Minas Gerais is the second most populous state in Brazil with a total population of 20,539,989 inhabitants [31]. It is located in the Southeast macro-region, and despite the increased number of ophthalmologists, inside of the state there is an important unbalance of personal and material resources in the health care system by microregions. Furthermore, Minas Gerais’ ethnical distribution (self-declared) in the last census showed that the state population is composed by 46.76% brown (multiracial), 41.08% white, 11.84% black, 0.16% indigenous and 0,15% east Asian population, portraying the multi-ethnic character present in Brazil [32]. Thus, a study that effectively used an AI-based tool such as the fluid monitor in a real-world sample from a this Latin population highlights the importance of testing newly algorithms worldwide.

This cohort is composed of patients in a treat-and-extend regimen, whereof 41.5% were non-treatment-naïve. Visual acuity at baseline is the most important predictor to BCVA in a long term follow-up, however, retinal fluid is still the most important anatomical biomarker in visual and late-stages outcomes, as well as for retreatment decisions in nAMD [33]. Thus, the volume, location, and its fluctuation play an important role in disease progression [16]. The results from our analysis demonstrated that higher fluid volumes in all three compartments were significantly corelated with worsening BCVA, noting that SRF was only significant when this fluid was also higher in the centre 1 mm of the macula. According to the literature, IRF and PED have been more associated with visual impairment. Yet, the topographic distribution of fluids with IRF more concentrated in the central fovea and SRF more distributed across the macular region may contribute to this statement [34]. A previous analysis conducted in the Caucasian population of the FRB! Zürich dataset showed that only IRF was associated with worse BCVA, however in the cited study fluid volumes were quantified after initial treatment and fluid volumes in this present cohort is significantly higher [20]. Furthermore, individual responses to available treatment needs to be always taken into consideration.

Regarding SRF behaviour there is an open question if a correlation with better visual outcomes is pertinent [35, 36], or even if a residual tolerable volume threshold can be settle. Previous analysis using data from the FLUID study showed that tolerating SRF had similar BCVA after 24 months of follow up than those treated in order to dry out the fluid [37]. On the other hand, a post hoc analysis also in the FLUID study data using the SRF-tolerant arm showed that 50% of the eyes with residual SRF increased the volume in the central 1 mm at the consecutive visit. The increase in SRF in the central 1 mm was correlated with significant decrease in BCVA in the next visit when compared with the non-tolerant group [38]. In addition, SRF fluctuation was found to be correlated with worse photoreceptor integrity in the OCT on the analysis from the OCTAVE clinical trial dataset, and there is a positive correlation between photoreceptor integrity and visual outcomes [39]. 

The limited period of follow-up associated with the exclusion criteria of presence of MA and SF in the central 6 mm area at baseline likely underestimates the rate of MA and SF development in this cohort when compared to the literature [40–42]. Furthermore, treat-and-extend regimen usually presents higher treatment frequency than pro-re-nata (PRN) which could also be associated with lower rates of these late-stage outcomes. Our results showed that higher IRF and PED volumes in the central 1 and 6 mm of the macula were associated with increased risk of developing SF. SRF was also associated with SF, but only when considering the 6 mm area. Fluid location may play a role in this finding since, as mentioned, SRF is often more present parafoveal than centrally [43]. Higher fluid volumes in any compartment did not show statistically significant correlation with MA development. MA pathophysiology remains unclear, however, previous findings showed that almost 100% of eyes with previous non-foveal atrophy will evolve to central atrophy over 10-years of follow-up [41]. These findings demonstrates the multifactorial components of MA development, possibly including genetic factors.

In this study we have founded a mean number of injections per eye of 5.9 over 12 months and 13.6 over 24 months. A previous report using DATASUS database showed a way lower number of 2.37 (1.35–3.43) as an overall annual median of injection in Brazilian public health care from 2014 until 2020 [11]. It is important to state that although anti-VEGF drugs were approved by the Brazilian federal agencies since 2007, only in 2018 the treatment was incorporated into the guideline of the Brazilian public health system, which surely impacted the median reported in this database over the years. Furthermore, if the supplementary system numbers were included in this analysis, the real mean number of injections in the Brazilian population per year would significantly increase and probably get closer to what was founded in this present study, which is also consisten with the literature worldwide.

In a daily basis clinical practice, anti-VEGF treatment can follow monthly, treat-and-extend or PRN regimens. In the PRN regimen, the decision is mainly driven by the dichotomous parameter of presence or absence of retinal fluids. In this study, higher SRF volume proved to be the most important driving force for an increased number of injections after one and two years. Previous studies from our group have also shown that SRF was the most important anatomic biomarker for predicting treatment need [19, 27], despite not being the most impactful fluid compartment in worse anatomical and visual outcomes. Certainly, IRF is actively treated in all regimens, since is the fluid compartment with the strongest correlation with worse BCVA and late-stage outcomes. Nevertheless, it seems to present a faster response to anti-VEGF treatment. Until now, PED is not an isolated target to anti-VEGF therapy, despite some previous studies have shown an anatomical response to the treatment and possible visual improvement [44]. Our study showed an association of higher volumes of PED with worse visual outcomes and faster development of SF, however further prospective studies are necessary to address this question.

The ageing population has continuously increased AMD prevalence and, consequently also increased anti-VEGF intravitreal injections demand [6]. Despite all effort in the nAMD research subfield, the available treatments demand continuous monitoring and injections, which means a high burden for the patient and for the health care system. The DATASUS report showed that OCT exams and anti-VEGF procedures were mainly done in the Southeast macro-region (87%). As previously stated, the higher population concentration and higher density of ophthalmologists in this area, certainly contributes to this finding [12, 31]. 

This study has inherent limitations for being a retrospective analysis performed on real-world patients and with only 2-years duration which is however in accordance with many randomized controlled trials. First, treatment was not standardized, despite mainly followed a treat-and extend regimen. Second, the images were not acquired specifically for this analysis, but as a clinical routine, thus number of B-scans are not uniform. However, a minimum of 19-Bscan was performed, which showed to be reliable for fluid quantification. Finally, there are other biomarkers such as SHRM and ellipsoid zone segmentation with a strong impact on visual acuity outcomes in AMD, which should be considered for future investigations in a prospective manner.

The possibility of monitoring patients using a real-time automated tool and specially assisting non-retinal specialists’ ophthalmology doctors can shorten some gaps regarding the health care unbalance between regions. This study investigates the multifaceted landscape of nAMD in a tertiary center in the Southeast Brazil, considering the country’s epidemiology and particular context, pointing the potential benefits of AI-driven technologies on patient’s care. Further studies that highlight the significance of using newly validated technologies, especially deep learning algorithms across diverse populations worldwide will be of interest.

## Data Availability

No datasets were generated or analysed during the current study.
